# Optimists and Realists: A Latent Class Analysis of Students Graduating from High School during COVID-19 and Impacts on Affect and Well-Being

**DOI:** 10.3390/ijerph20032120

**Published:** 2023-01-24

**Authors:** Ana Zdravkovic, Abby L. Goldstein

**Affiliations:** Applied Psychology and Human Development, Ontario Institute for Studies in Education, The University of Toronto, Toronto, ON M5S 1V6, Canada

**Keywords:** COVID-19, well-being, adolescents, life satisfaction, stress, affect, latent profile analysis

## Abstract

The Novel Coronavirus Disease (COVID-19) pandemic has had profound effects on physical and mental health worldwide. Students transitioning out of high school were uniquely impacted at the onset of the pandemic, having missed the opportunity to properly mark the end of their final year in the K-12 school system. The adverse effects of this loss on this population are still unknown. The purpose of the current study was to examine stress, wellbeing, and affect in a sample of 168 students (*N* = 168; *M*age = 17.0, *SD* = 0.46; 60% female; 40% male) who were completing their final year of high school during the early stages of the pandemic when emergency stay-at-home orders were in place. Participants completed an online survey assessing the impact of COVID-19 on their life satisfaction (pre-COVID19, during COVID-19, and anticipated five years from now), stress, positive affect, and negative affect. Latent class analysis (LCA) was used to create classes of participants based on their responses to the pandemic. A two-subgroup solution provided the best model for the life satisfaction outcome variable. Subgroup 1, *optimists*, comprised 24% (*N* = 40) of the sample and reported high life satisfaction ratings one year prior to COVID-19 and a slight decrease in life satisfaction during COVID-19, and they anticipated an increase in life satisfaction 5 years from now. This group was characterized by low stress, low negative affect, and high positive affect during the pandemic. Subgroup 2, *realists*, comprised 76% of the population (*N* = 128) and experienced similarly high retrospective ratings of pre-COVID life satisfaction but a larger decrease in life satisfaction during the pandemic and a smaller increase in five years. The realist group was characterized by low positive affect, high stress, and high negative affect during the pandemic. The findings suggest that during the pandemic, certain subsamples of adolescents had greater difficulty in managing this transitional period and experienced changes in mood and well-being (i.e., affect, stress) as compared to other adolescents (i.e., *optimists*). Future research should investigate the characteristics and coping mechanisms that are instrumental for increasing life satisfaction and positive affect while lowering stress in this population.

## 1. Introduction

In December 2019, the Coronavirus disease (COVID-19) was first reported in Wuhan, central China [1,2]. The virus rapidly spread and was declared a global pandemic by the World Health Organization (WHO) in March 2020 [3]. Following recommendations from the WHO and Centers for Disease Control (CDC), the Canadian government implemented several public health measures, including a stay-at-home order that effectively cut off non-essential personal contact [4,5]. This shift created significant stress on people worldwide, impacting their employment status, well-being, and mental health [1,6,7,8,9].

In Canada, youths aged 14 to 28, with and without preexisting mental health challenges, experienced an increase in mental health concerns in the early phases of COVID-19 [10]. Recent research has further highlighted the mental health implications of COVID-19 on youths, and researchers have implied that mental health symptoms will likely continue to increase globally due to the pandemic [1,11]. Furthermore, researchers have suggested that, relative to older adults, young adults were particularly vulnerable to increased distress during the pandemic [12]. This is not surprising, given that this stage of life is already a time of increased instability, uncertainty, and critical transitions [13,14].

One of the impacts of COVID-19 for youths was on learning and school-related activities, including the cancellation of extra-curricular and social events. Canadian students making the transition out of high school faced multiple losses in the early stages of the pandemic. Instead of marking the end of Grade 12 with their school communities, students finished the year online, away from peers, and without the typical events that mark the end of a K-12 career, including final exams, prom, and graduation. Previous researchers have found that home confinement during the early stages of the pandemic was associated with greater negative impacts on mental health [15]. For adolescents, interactions with peers are pivotal [13,16,17,18] and promote positive adjustment [19], which are crucial for mental and physical health [20]. The stay-at-home mandates in 2020 disrupted many of these relationships, leading to significant impacts on life satisfaction and wellbeing [21,22,23]. A survey of 1054 Canadian adolescents (*M*age = 16.68) found that individuals were most concerned about the impact on their schooling, followed by general concerns about the COVID-19 crisis and not feeling connected to friends [24]. As such, this is a population that requires attention regarding the long-term impacts of the pandemic on their overall well-being.

Subjective well-being is an important aspect of positive adjustment and functioning, consisting of several different components. Hedonic well-being comprises affective [23,25] and cognitive components [23,26,27]. Life satisfaction, a cognitive component, has been well researched in the literature and has been found to be related to increased happiness and hope, and decreased depression [28], whereas low life satisfaction is related to negative feelings and emotions [29]. High positive and low negative affect are two other key aspects of well-being [30]. Taken together, individuals are more likely to experience happiness when both positive affect and life satisfaction are high [31]. In contrast, high negative affect and low positive affect are related to elevated psychological distress [32]. Adverse events that are out of an individual’s control, such as COVID-19, are expected to have negative impacts on well-being via lower positive affect, higher negative affect, and reduced life satisfaction [33]. Research by Maher et al. (2021) found that, when compounded with the introduction of stressors, perceptions of positive affect decreased during COVID-19, whereas negative affect increased in a college student sample [34]. Zacher and Rudolph (2021) found that subjective well-being decreased significantly in the early months of the pandemic (between March and May 2020), as did positive affect (surprisingly, negative affect also decreased) [35]. Although these reductions in well-being are expected, what is less known is whether adolescents who were experiencing multiple losses amidst a time of uncertainty and transition still believed that things would improve in the future. Typically, there is an upward trajectory in ratings of life satisfaction where individuals rate their current satisfaction as better than their past, but believe they will be even more satisfied in the future [25]. Even during COVID-19, adults in the US believed that their life satisfaction would increase in the future, despite their current life satisfaction being lower than usual [36]. Although youth and emerging adults are an optimistic group who believe that their future is bright and expect greater life satisfaction than their parents [37], it was not clear that this same optimism would be upheld during COVID-19 given the multiple losses that they experienced. This is important, as optimism, or hope for the future, are associated with better wellbeing over time [38,39], whereas pessimism is associated with greater stress [40].

To better understand the impact of the pandemic on youth optimism regarding their current and future life satisfaction, we used a Latent Class Analysis (LCA). LCA has been used as a classification method for understanding patterns of mental health and well-being [41,42], as it allows for classes, or groups, to be created based on patterns in observed item responses that measure an underlying latent construct [43]. We applied LCA to the current study, where we anticipated that different patterns of life satisfaction (past, present, future) would arise and be linked to different well-being outcomes. Current research suggests that life satisfaction among Canadians declined during the pandemic, with youths experiencing one of the greatest declines [44]. What is not yet known is whether youths who were graduating from Grade 12 in 2020 experienced decreases in life satisfaction during COVID-19, whether youths experienced different life satisfaction trajectories during the pandemic (i.e., they anticipated a more difficult future), and what other facets of well-being were associated with trajectories of life satisfaction during this time.

Using an LCA approach, we anticipated that different subgroups would emerge based on retrospective, current, and prospective life satisfaction ratings. Based on the literature, we expected two subgroups to emerge; one to follow the typical trajectory of increasing life satisfaction overtime [13,45,46], and a second to follow a COVID-19-impacted trajectory with decreased life satisfaction that will persist past the pandemic [47]. In addition, we anticipated that these two groups would differ on other indicators of well-being, with one group (optimistic/typical trajectory) showing lower levels of stress, higher positive affect, and lower negative affect, and the other group (realistic/COVID-19-impacted trajectory) showing greater stress, lower positive affect, and higher negative affect during the pandemic.

As the pandemic continues to impact millions of people worldwide, there are widespread concerns about its toll on youth and emerging adult mental well-being and life satisfaction [47,48,49]. However, there is limited work examining life satisfaction trajectories, particularly among youths who were transitioning out of high school during the initial pandemic lockdowns. The goal of this research was to expand on previous research findings by exploring the life satisfaction trajectories have emerged among this population and the well-being indicators that further describe membership in these trajectories.

## 2. Materials and Methods

### 2.1. Participants

The current sample consisted of 168 Grade 12 students (*M*age = 17.0, *SD* = 0.455; 60% female, 40% male) who were completing their final year of high school in Ontario in 2020. Participants completed an online survey assessing life satisfaction, perceived stress, and positive and negative affect. The online surveys were created via Qualtrics [50]. Participants were recruited from across Ontario and were not limited to a single school. The examiner primarily shared fliers across social media platforms (i.e., Facebook groups and pages, Twitter, etc.) and used word of mouth to recruit participants. A URL link was sent to participants who were deemed eligible for the study by reviewing their valid student ID cards. The inclusion criteria for participants were adolescents (age ≥ 18) who were in their final year of high school during March 2020 and who were willing to participate in this study. Informed consent was gathered via the online Qualtrics survey. The study protocol received ethical approval by the University of Toronto. Socio-demographic information can be found in Table 1.

### 2.2. Measures

A demographic questionnaire including specific questions related to the impacts of COVID-19 was used in this study. Questions included age, gender identity, ethnicity, living arrangements during the pandemic outbreak, employment, and employment change during the pandemic, as well as potential change in plans after high school due to the pandemic.

Perceived Stress Scale (PSS) [51].

The PSS was adapted and used to assess stress during the first wave of the COVID-19 pandemic (e.g., “during the COVID-19 pandemic, how often did you feel things were going your way?”). The PSS is a 10-item scale that captures the degree to which situations are perceived as stressful on a scale from 0 = never to 4 = very often [52]. Ratings were averaged across items such that higher scores represented greater perceived stress. The PSS-10 has demonstrated strong internal consistency and convergent and divergent validity among university students [53].

Satisfaction with Life Scale (SWLS) [54].

The SWLS was used to capture life satisfaction, an important aspect of wellbeing. Participants rated their life satisfaction on a 7-point scale (from 1 = strongly disagree to 7 = strongly agree). They were asked to retrospectively rate their life satisfaction last year, their current life satisfaction (during COVID-19), and their predicted life satisfaction in five years.

Positive and Negative Affect Schedule Short Form (PANAS-SF) [32].

To measure positive and negative affect, participants completed the 20-item PANAS-SF, rating their feelings of both positive (e.g., excited, enthusiastic, proud) and negative (e.g., distressed, irritable, upset) affect during COVID-19 on a scale from 1 = very slightly to 5 = extremely. Scores range from 10–50 for both positive affect (PA) and negative affect (NA), with the lower scores representing lower levels of positive/negative affect. The PANAS has demonstrated a good internal reliability that was consistent with the scores obtained, ranging from 0.86 to 0.90 for PA and 0.84 to 0.87 for NA.

### 2.3. Data Analysis

A Latent Class Analysis (LCA) with MPlus 7.4 [55] using the maximum likelihood ratio (MLR) [56,57] and 500 iterations was conducted to determine the number of latent classes that could be extracted from our data based on participants’ self-reported life satisfaction pre-COVID-19, during COVID-19, and in five years. LCA is a person-centered method that reveals homogenous subgroups of individuals that vary in the expression of different features [58]. This process is appropriate for cross-sectional mixture modeling when exploring phenotypic profiles [59] and understanding developmental heterogeneity in populations [60,61,62]. Many researchers have published papers encouraging the use of LCA in mental health and developmental research as it properly addresses pivotal research questions in the field [63].

LCA models with 1–4 classes were tested. Three indicators were used to determine the best model fit. The Lo–Mendell–Rubin (LMR) test was used to compare the model fit of the two-, three-, and four-subgroup solutions that had similar covariance matrix parameterizations [55]. To compare the model fit of the models with different covariance matrix parameterizations, the Sample-Size-Adjusted Bayesian Information Criterion (SABIC) [64] and the Bayesian Information Criterion (BIC) [56] were used [42]. Based on the results of the LCA analyses, class membership variables were created for adolescents in the sample to examine whether wellbeing classes were associated with stress, positive affect, and negative affect during COVID-19.

## 3. Results

### 3.1. Determining the Model of Best Fit

Pearson correlations revealed significant correlations between stress, all measures of well-being, positive affect, and negative affect, as shown in Table 2.

The LCA started with one class. Additional classes were added, and the model fit was assessed until the optimal number of classes was found. The Bootstrap Likelihood Ratio Test (BLRT) was performed to compare the model fit between the number of classes. The classification performance of the solution was estimated by discriminant analysis and a k = 10-fold cross-validation based on Gaussian finite mixture modeling. Specifically, we compared two-, three-, and four-subgroup models for each group to determine the best solution. In Model 1, covariances were specified to be zero (i.e., indicators were not specified to be related to one another), variances of the indicators were constrained to be equal across subgroups, and distal outcomes were fixed by fixing the variances and slopes to zero to allow the trajectories to vary within the groups around different means [65]. In Model 2, covariances were specified to be zero. In Model 3, covariances were specified to be zero, but variances were allowed to vary within and across subgroups. In Model 4, covariances were specified to be zero, but variances were allowed to vary within and across subgroups. The fit statistics are summarized in Table 3.

Model 3 yielded the lowest AIC, BIC, and SABI values [66]. However, the LMR log-likelihood ratio test for Model 3 yielded non-significant *p* values and a weaker entropy, suggesting that the three-group solution should be rejected in favor of the two-group solution. When the entropy values approached 1.0, there is evidence that a clear delineation between the latent classes existed [67,68]. The rationale for not simply relying on the AIC and BIC values was that AIC tends to overestimate the number of classes present, while BIC may underestimate the number of classes present, particularly in smaller sample sizes such as that of the present study [69]. Further, the four-class model was rejected in favor of the two-group solution because it was not significantly better than the three-group solution. Therefore, a two-class solution was selected.

### 3.2. Latent Classes in the Life Satisfaction Groups

**Figure 1:**
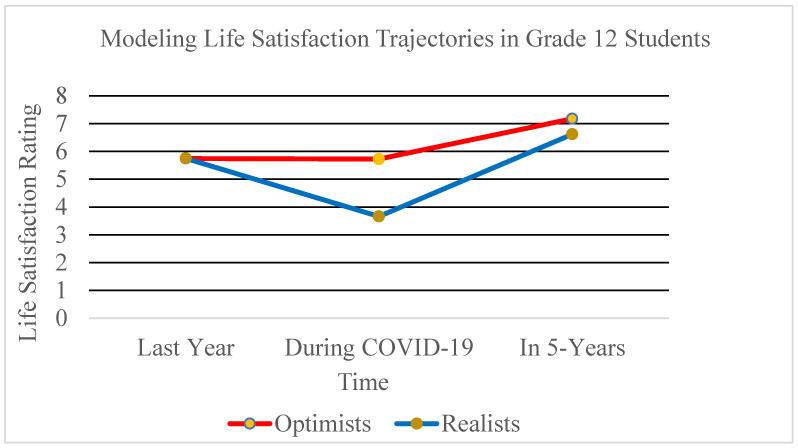
Latent classes of life satisfaction over time.

Examination of the fit statistics showed that a two-subgroup solution provided the best model. The subgroups had a good proportion of participants in each, making it the most interpretable and parsimonious [62], further supporting the choice for a two-class model. The deteriorating sample size was attributed to participants who did not complete all the questionnaires in the online survey and thus were not included in analyses. Our LCA revealed two distinct latent groups. As shown in Figure 1, the class labelled Class 1 (optimists; 24% of the sample) reported high life satisfaction ratings one year prior to COVID-19 (*M* = 5.74, *SE* = 0.305), with a slight decrease in life satisfaction during COVID-19 (*M* = 5.73, *SE* = 0.180). This group rated their expected life satisfaction in 5 years as increasing even further (*M* = 7.18, *SE* = 0.183), thus receiving the label of optimists. The second group, Class 2 (realists; 76% of the sample), experienced similarly high retrospective ratings of their past year life satisfaction (*M* = 5.75, *SE* = 0.126), but they experienced a much larger decrease in life satisfaction ratings during the pandemic (*M* = 3.67, *SE* = 0.116). Their predictions of life satisfaction in five years also increased, but not as much as Class 1 (*M* = 6.62, *SE* = 0.129).

### 3.3. Covariate Analysis

**Figure 2:**
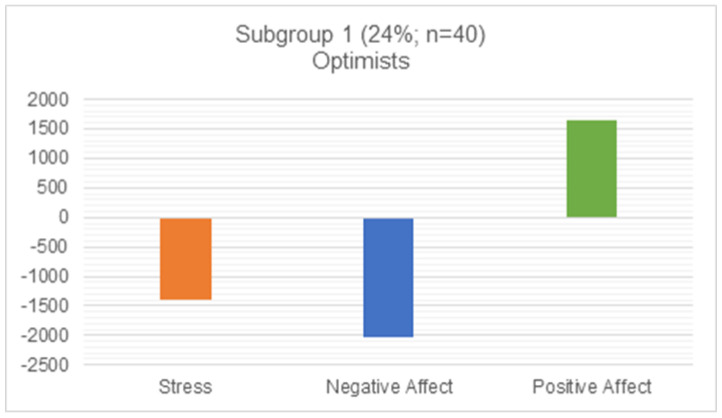
Class 1 relationship with stress, negative affect, and positive affect.

**Figure 3:**
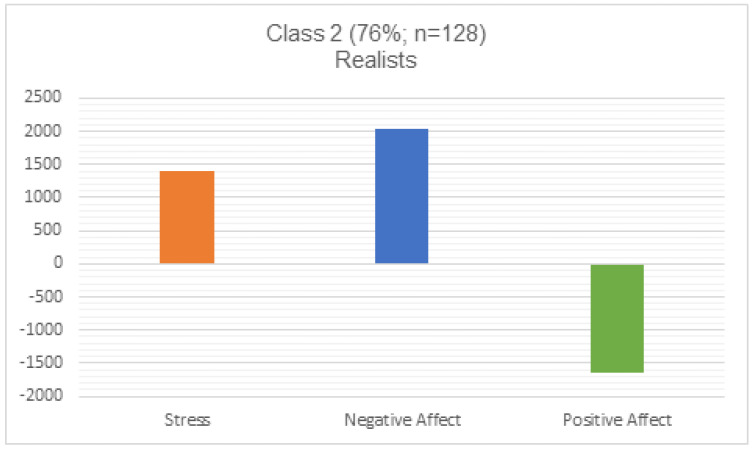
Class 2 relationship with stress, negative and positive affect.

To examine the relationship between life satisfaction trajectories and other facets of well-being (stress, negative affect, and positive affect), we conducted a post hoc multinomial linear regression analysis. Our analysis reflected that, compared to Class 2 (realists), individuals in Class 1 (optimists) experienced lower stress, lower negative affect, and higher positive affect during the first wave of the pandemic. Individuals in the realist class reported lower positive affect but higher stress and negative affect (see Figure 2 and Figure 3).

## 4. Discussion

The current study sought to assess the trajectories of life satisfaction (past, current, and future assessed cross-sectionally) among Canadian Grade 12 students (i.e., those finishing high school) during the first wave of the COVID-19 pandemic. We focused on this group due to the many losses they experienced during the pandemic, which interfered with their final months of high school and led to cancelled proms, graduation ceremonies, and an uncertain transition into postsecondary education. Using LCA, we found that two subgroups emerged from the sample. The subgroups were similar regarding their ratings of life satisfaction pre-pandemic. In addition, both groups showed the typical pattern of anticipating that things would improve in the future (i.e., the next five years), reflecting a degree of resilience and openness to increasing opportunities despite the many losses they experienced. What differed between the groups were their experiences during COVID-19 and the degree to which they anticipated improvements in life satisfaction post COVID-19. The optimists experienced only a small decline in life satisfaction during the pandemic, appearing more resilient to the impacts of COVID-19, and felt as though they would bounce back to optimal life satisfaction in the future. The second subgroup, the realists, seemed more impacted by the pandemic, feeling less satisfied with life during this time and less optimistic of their future.

These two groups were also distinguished by their experiences of well-being during the pandemic. Participants in the optimists group showed resilience in several ways: they experienced greater positive affect, lower negative affect, and had lower stress during the pandemic. Those in the realist group experienced more challenges to their well-being. Their stress was higher, their negative affect was higher, and their positive affect was lower. This is consistent with other research conducted during the COVID-19 pandemic, which found that college students experienced higher levels of negative affect and lower levels of positive affect [34]. Our findings indicate that the impacts of the pandemic on well-being and life satisfaction were not homogenous across this population. Although participants were not necessarily pessimistic about the future, a subgroup of individuals—the realists—experienced dampened life satisfaction and were more susceptible to negative impacts on their well-being, including greater stress and negative affect. This is consistent with research from Magson and colleagues (2021) who found that adolescents experienced significant decreases in life satisfaction during the pandemic [47]. Our findings, however, were unique in that they spoke not only to well-being during the pandemic but also to anticipated well-being.

Certain protective factors likely helped a subset of participants fare better during the pandemic. Li, Dou, and Liu (2022) conducted a Latent Profile Analysis (LPA) to understand positive changes among adolescents during the pandemic and found that three subgroups emerged: one with limited positive changes (33.3%), one with partial positive changes (49.5%), and one with overall strong positive changes (17.2%) [70,71]. Adolescents in the overall strong positive changes profile had better mental health and well-being outcomes than those in the other two profiles. They also had greater resilience, which has been found to lead to more positive outcomes for adolescents. Our findings were similar; students with higher resiliency, in the form of higher positive affect, lower negative affect, and lower stress, fared better regarding life satisfaction during the pandemic and anticipated higher life satisfaction in 5 years. This group displayed optimism, a quality that has been linked to increased life satisfaction. Optimism is positively correlated with coping strategies that help to regulate negative emotions and stressors [72], manage adversity more efficiently [73], and predict successful coping with significant life stressors [74,75]. Researchers have also indicated that optimism is adaptive when dealing with long-term stressors [71]. One risk associated with optimism is unrealistic optimism, where predictions made by people are more optimistic than is objectively warranted by the evidence [76]. For example, in the context of COVID-19, when people think about the future, they tend to underestimate their chances of contracting COVID-19 and becoming seriously ill. Bortolli et al. (2015) suggest that optimistic thinking may not actually be as beneficial to people as we may think; it has the potential to lead to significant psychological costs (i.e., increased risk-taking behaviors). Rather, realistic beliefs and expectations can be catalysts for well-being and good functioning. As such, even though our research revealed that the realist subgroup reported lower life satisfaction, paired with low positive affect, high negative affect and stress, they may in fact have more “realistic” predictions of the future, which might subsequently lead to increased well-being and positive functioning [75,76].

### Limitations

While this study provided new information regarding the impact of COVID-19 on students who were finishing Grade 12 during the initial onset of the pandemic, it was not without limitations. Firstly, the project drew from prospective and retrospective ratings of life satisfaction, which may not reflect actual experiences across time. Longitudinal data would provide further support for the optimist/realist subgroups, as we cannot know with certainty that having a more positive outlook on future life satisfaction will in fact lead to actual increases in life satisfaction and well-being, nor can we track the potential future payoff of these predictions. In addition, we did not measure pre-existing mental health concerns, and previous researchers have found that responses to COVID-19 varied as a function of mental health status [77]. Our data was collected early in the pandemic when it was not yet known that individuals with and without mental health concerns would experience different COVID-19 impacts. Future research on pandemic stressors should consider pre-existing mental health concerns, along with personality and current stress, to better understand changes from the introduction of a significant stressor. Researchers should also assess the availability of resources for students to implement programming more effectively for youth and emerging adults who are entering post-secondary education during times of uncertainty. Our research sample was relatively small and predominately Caucasian and included participants within a restricted age range. Further, the data were collected within a single province in Canada (Ontario). Thus, our findings may not be generalizable to high school graduates from other countries who experienced different rates of infection and severity of government-imposed restrictions. Finally, while we did not find any significant gender differences in our data, future research should consider different variables that might further define group membership (i.e., demographic, culture, SES). For example, previous research indicates that gender may play a role [78]. This will aid in developing programming for specific groups that experienced significant negative impacts from the pandemic.

## 5. Conclusions

The goal of the current study was to assess the impacts of the global pandemic on a subgroup that has not yet been widely examined. We applied a person-centered approach to understand the emerging subgroups of Grade 12 students in terms of their retrospective, current, and predicted life satisfaction during unprecedented times. Overall, we found that this approach revealed two subgroups that could be distinguished by their experiences of life satisfaction during COVID-19. Namely, students in the *realists* subgroup experienced significantly thwarted life satisfaction compared to the *optimists*, who experienced a much smaller decline in life satisfaction during the pandemic. Further, these groups differed in their experiences of stress and affect, where the *realists* experienced higher stress and negative affect, but lower positive affect, and the *optimists* experienced lower stress and negative affect, but higher positive affect. Thus, it is clear that one subgroup of this sample fared much better during the pandemic, which was a significant adverse event. Subsequent research should use longitudinal data to understand what protective factors actually helped students to experience better well-being and more positive future functioning (i.e., adaptive coping behaviors, social support). This will help researchers to develop programming that helps students foster these skills to mitigate their stress and manage adverse events.

## Figures and Tables

**Table 1:** Demographic characteristics and impacts of COVID-19 for participants (*n* = 178).

Variables	(%)
**Gender**	
Male	(60%)
Female	(40%)
**Ethnicity**	
Caucasian	(20%)
East Asian	(16%)
South Asian	(16%)
Southeast Asian	(2.3%)
Filipino	(3.5%)
West Indian	(2.3%)
Black	(4.7%)
Latin American	(0.6%)
Arab	(4.7%)
Other	(3.5%)
**Living Arrangements in March 2020**	
Living at home with their parents	(94%)
Living with friends or roommates	(3.4%)
Living alone	(1.7%)
Living with other family	(0.6%)
**Employment Status Prior to Onset of COVID-19**	
Employed	(41%)
Unemployed	(59%)
**Employment Status Following Onset of COVID-19**	
Still working, but less	(41%)
Temporarily laid off	(37%)
Delaying the start of a new job	(1.4%)
Looking for work	(11%)
Lost a new job that was supposed to start in the future	(1.4%)
No longer looking for work	(8.2%)
**Impact of COVID-19 on Plans Following Highschool**	
No change in plans	(81.9%)
Plans have changed	(18.1%)
Unsure about future plans	(50%)
Planning to work	(23.3%)
No longer planning to attend university in Canada	(3.3%)
No longer planning to attend university outside of Canada	(10.0%)
Taking a gap year	(10%)
Staying home and caring for a family member	(3.3%).

**Table 2:** Descriptive statistics: means and correlations.

Measure	M	SD	1.	2.	3.	4.	5.	6.
1. Life Satisfaction Last Yr	5.74	1.53	1.000					
2. Life Satisfaction COVID-19	4.19	1.54	0.075 *	1.000				
3. Life Satisfaction 5 Yrs	6.78	1.53	0.170	0.084 **	1.000			
4. COVID-19 Stress	20.39	6.55	0.050 *	−0.461 **	−0.131 *	1.00		
5. Positive Affect	28.95	7.50	0.447 **	0.509 **	0.628 **	−0.364 **	1.000	
6. Negative Affect	25.57	8.15	−0.365 *	−0.319 **	−0.133 *	0.684 **	−0.115 *	1.00

*Note:* Life Satisfaction Last Yr = life satisfaction last year; Life Satisfaction COVID-19 = life satisfaction during COVID-19; Life Satisfaction 5 Yrs = life satisfaction in five years; COVID-19 Stress = perceived stress during COVID-19; Positive Affect = positive affect during COVID-19; Negative Affect = negative affect during COVID-19. ** Correlation is significant at the 0.01 level (two-tailed) * Correlation is significant at the 0.05 level (two-tailed).

**Table 3:** Fit indices from one-class, two-class, three-class, and four-class mixture models.

Model				
	1-Class	2-Class	3-Class	4-Class
H_0_	−3243.232	−813.162	−781.876	−766.438
AIC	6518.464	1656.324	1611.752	1598.875
BIC	6569.283	1703.183	1686.728	1701.966
SABIC	6518.613	1655.690	1610.738	1597.481
ENT		1.000	0.871	0.884
VLMRLRT *P*		0.000	0.6538	0.6755
LMR p for K−1		0.000	0.6538	0.6755
*N*	177	168	168	168

*Note:* AIC = Akaike information criterion; BIC = Bayesian information criterion; ABIC = adjusted Bayesian information criterion; ENT = entropy; VLMRLRT *P* = Vuong–Lo–Mendell–Rubin likelihood ratio tests; LMR; Lo–Mendell–Rubin.

## Data Availability

The data presented in this study are available on request from the author who carried out the analyses (A.Z.).
